# The New Society of Hahnemannians

**Published:** 1896-01

**Authors:** 


### THE NEW SOCIETY OF HAHNEMANNIANS.

A meeting of the Hahnemannians of Brooklyn was held on Saturday evening, January 25th, 1896, at the residence of Dr. Stuart Close, 641 Willoughby Avenue, for the purpose of forming an organization, which should be the means of bringing about closer personal relations between members, mutual improvement and encouragement, and the advancement of pure Homœopathy in Brooklyn.

The following physicians were present at the first meeting : Dr. B. L'B. Baylies, Dr. Alice Boole Campbell, Dr. John B. Campbell, Dr. Anna Carman, Dr. Stuart Close, Dr. B. Fincke, and Dr. F. H. Lutze.

The meeting was called to order by Dr. Close, and discussion of the principles and methods of the new society proceeded.

The Brooklyn Hahnemannian Union was the name decided upon.

Meetings will be held on the last Saturday evening of each month at 641 Willoughby Avenue until further notice.

A chairman will be elected at the close of each meeting to preside at the next meeting, and to prepare and present a paper or subject for discussion. Members will be notified two weeks in advance of the subject chosen by the chairman for the next meeting. The only permanent officer will be a secretary. Mrs. Stuart Close was elected to fill this position.

Meetings are to be as nearly informal as is consistent with the orderly conduct of business, and a spirit of charity, freedom, fraternal feeling, and love for the noble art of Homœopathy cultivated.

After the business of organization had been completed, Dr. Close read a paper on "The Artistic Spirit of Medicine," which was cordially received and recommended for publication in THE HOMŒOPATHIC PHYSICIAN.

General discussion of the paper offered, and narration of interesting cases by Drs. Baylies and Fincke followed.

A repast was served by the hostess, and the meeting adjourned, after having elected Dr. Baylies chairman for the ensuing meeting.

